# Multiple Pathways of Plasmid DNA Transfer in *Helicobacter pylori*

**DOI:** 10.1371/journal.pone.0045623

**Published:** 2012-09-20

**Authors:** Stefanie Rohrer, Lea Holsten, Evelyn Weiss, Mohammed Benghezal, Wolfgang Fischer, Rainer Haas

**Affiliations:** 1 Max von Pettenkofer-Institut für Hygiene und Medizinische Mikrobiologie, Ludwig-Maximilians-Universität, München, Germany; 2 Ondek Pty Ltd and H. pylori Research Laboratory, Microbiology and Immunology, University of Western Australia, Nedlands, Australia; Centre National de la Recherche Scientifique, Aix-Marseille Université, France

## Abstract

Many *Helicobacter pylori* (*Hp*) strains carry cryptic plasmids of different size and gene content, the function of which is not well understood. A subgroup of these plasmids (e.g. pHel4, pHel12), contain a mobilisation region, but no cognate type IV secretion system (T4SS) for conjugative transfer. Instead, certain *H. pylori* strains (e.g. strain P12 carrying plasmid pHel12) can harbour up to four T4SSs in their genome (*cag*-T4SS, *comB*, *tfs3*, *tfs4*). Here, we show that such indigenous plasmids can be efficiently transferred between *H. pylori* strains, even in the presence of extracellular DNaseI eliminating natural transformation. Knockout of a plasmid-encoded *mobA* relaxase gene significantly reduced plasmid DNA transfer in the presence of DNaseI, suggesting a DNA conjugation or mobilisation process. To identify the T4SS involved in this conjugative DNA transfer, each individual T4SS was consecutively deleted from the bacterial chromosome. Using a marker-free counterselectable gene deletion procedure (*rpsL* counterselection method), a P12 mutant strain was finally obtained with no single T4SS (P12ΔT4SS). Mating experiments using these mutants identified the *comB* T4SS in the recipient strain as the major mediator of plasmid DNA transfer between *H. pylori* strains, both in a DNaseI-sensitive (natural transformation) as well as a DNaseI-resistant manner (conjugative transfer). However, transfer of a pHel12::*cat* plasmid from a P12ΔT4SS donor strain into a P12ΔT4SS recipient strain provided evidence for the existence of a third, T4SS-independent mechanism of DNA transfer. This novel type of plasmid DNA transfer, designated as alternate DNaseI-Resistant (ADR) mechanism, is observed at a rather low frequency under *in vitro* conditions. Taken together, our study describes for the first time the existence of three distinct pathways of plasmid DNA transfer between *H. pylori* underscoring the importance of horizontal gene transfer for this species.

## Introduction

*Helicobacter pylori* is a highly motile, microaerophilic, Gram-negative bacterium, resident in the gastric mucus layer of about 50% of the human population. Infection with *H. pylori* is a major cause of gastroduodenal disease, including chronic active gastritis, peptic ulcer disease, mucosa-associated lymphoid tissue (MALT) lymphoma and gastric carcinoma [1], [2]. A remarkable feature of *H. pylori* is its panmictic population structure, reflected by an extreme genetic heterogeneity, possibly resulting from frequent recombination events after import of small pieces of foreign DNA from other *H. pylori* strains during persistent or transient mixed infections [3]–[6]. Such an efficient DNA exchange has been attributed to the natural transformation competence of *H. pylori* [7], mediated by the *comB* type IV transport system [8]–[10], which is actually stimulated by DNA damage to trigger genetic exchange [11].

Besides natural transformation, bacterial conjugation is a further possible mechanism of lateral DNA transfer. Interestingly, about 50% of *H. pylori* isolates carry cryptic plasmids ranging between 2 and 100 kb in size [12]. Some isolates even carry multiple plasmids of different size; however, the role of these plasmids for *H. pylori* is not well understood. Chromosomal integration and excision of plasmid DNA after transfer from a donor into a recipient strain might be an alternative way to generate genome rearrangements [13].

Most *H. pylori* plasmids either replicate via the “rolling circle” mechanism [14], [15], or carry direct sequence repeats, so-called “iterons”, and replicate via the theta mechanism [16] (for review see [17]). A DNaseI-resistant, conjugation-like bidirectional chromosomal DNA transfer between *H. pylori* has been reported, but the mechanism has not been explored [18]. Conjugative transfer of indigenous plasmids between *H. pylori* strains, or even more unrelated bacterial species, has not yet been demonstrated.

Plasmids are generally classified as conjugative (autotransmissible) or mobilisable (transmissible only in the presence of a helper conjugative plasmid). In contrast to conjugative plasmids, which contain all the necessary genetic information to catalyse conjugative DNA processing and DNA transport, mobilisable plasmids lack part of this machinery. Mobilisable plasmids typically have an origin of conjugative transfer (*oriT*) and code for proteins involved in conjugative processing of DNA, such as *oriT*-specific relaxases and nicking accessory proteins [19].

**Figure 1:**
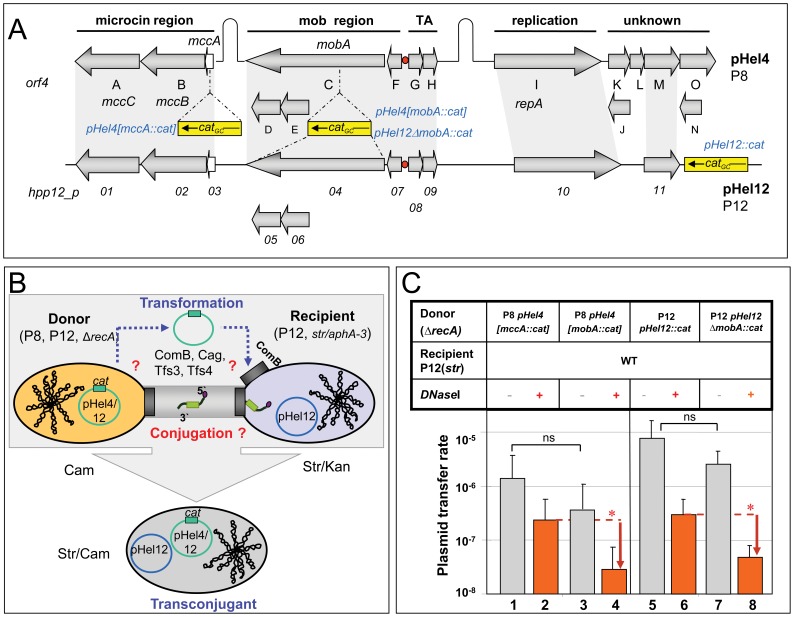
Structure of cryptic plasmids pHel4 and pHel12 and verification of their intra- and interstrain plasmid transfer. (A) Schematic map showing the gene content and basic functional regions of two cryptic plasmids pHel4 and pHel12. Both plasmids contain a microcin gene cluster with homology to *E. coli mccC7* (*orf4A*, *B*, *X*, or *orf01*, *02*, *03*). Genes *orf4C-F* and *orf04-orf07,* respectively, show homology to the *mob* region of colicinogenic plasmids. Genes *orf4G/H* and *orf08*/*09,* respectively, are related in sequence to the toxin-antitoxin system RelE-RelB (TA). The plasmid replicase is encoded by *orf4I* (pHel4) or *orf10* (pHel12). Both plasmids also carry a number of hypothetical genes (*orf4J-O*, *orf11*). The insertion of the *cat*_GC_ antibiotic resistance gene cassette into various *orfs* is shown. (B) Procedure of *H. pylori* co-cultivation to determine plasmid DNA transfer via natural transformation or conjugative processes. All T4SS (*cag*-PAI, *comB*, *tfs3*, *tfs4*) potentially involved in plasmid transfer between an *H. pylori* donor (P8, 12) and a recipient (P12) strain are indicated. Generally, natural transformation of the recipient strain by released plasmid DNA after lysis of the donor strain and conjugation processes are superimposed and are discriminated by adding of DNaseI. Plasmid transfer of pHel4[*mccA::cat*] or pHel12::*cat* from strain P8 or P12 into the P12 recipient strain was monitored by selection of the recipient strain (P12) via streptomycin (P12*^str^*) or kanamycin (*P12moeB::aphA-3*). To avoid the transfer of the chromosomal marker of the recipient (*str, aphA-3*) into the donor strain by natural transformation, the *recA* gene in the donor strain was deleted (P8Δ*recA::erm*, P12Δ*recA::erm*). (C) Inter- or intrastrain transfer of the cryptic plasmids pHel4[*mccA::cat*] or pHel12::*cat* from donor strain P8 or P12 into the recipient strain P12*^str^* in the absence (−) or presence (+) of DNaseI. Transfer rates were determined as the number of transconjugants/cfu/ml. Data shown are mean values of at least three independent experiments including standard deviations. * p<0.05, ns, not significant.

We have initially characterized two cryptic *H. pylori* plasmids of 10.9 and 18.2 kb in size named pHel4 and pHel5, respectively [13], and recently pHel12 [20], a 10.2 kb plasmid. They were assigned to the group of theta plasmids. In pHel4 and pHel12, a putative *mob* region was identified, showing best homologies to proteins MbeA, MbeB, MbeC and MbeD of colicinogenic plasmids, such as pColE1 [13], [21]. The MbeA protein of the ColE1 plasmid is an atypical relaxase, because it lacks two conserved histidine residues in the third conserved amino acid sequence motif (motif III) [22], present in most relaxases. MbeA, which reveals best sequence homology in the three conserved amino acid motifs (motif I, motif II and motif III) to the pHel4 relaxase [13], has been verified as a relaxase necessary for plasmid ColE1 mobilisation [23]. *H. pylori* plasmid pHel4 is 10.9 kb in size. In addition to its encoded replicase RepA, the plasmid contains a putative microcin operon, a putative mobilisation region (*mob*A) and a number of cryptic open reading frames (ORFs) with low homology to *H. pylori* chromosomal genes (Fig. 1A) [13]. Plasmid pHel12 is rather homologous to pHel4 in its gene content and primary sequence [20]. It carries a microcin region as well as a putative mobilisation region, but contains only a homologue of *orf4M* (HPP12_12), but no homologues of *orf4K*, *L*, *N* and *O*. We show here that cryptic plasmids of *H. pylori* can be transferred by at least three different mechanisms: natural transformation, mobilisation and a DNaseI-resistant alternate pathway.

## Results

### Generation of a Model System to Monitor Directional *H. pylori* Plasmid Transfer

Several plasmids carrying putative *mob* regions have been described for *H. pylori* [17]. They are candidates for a conjugative transfer between *H. pylori* strains. In this study, plasmids pHel4, present in *H. pylori* strain P8, and pHel12, present in strain P12 (Fig. 1A), were analysed for their transfer potential. The *comB* T4SS (*tfs2*) is involved in plasmid and chromosomal DNA uptake by natural transformation and is present in all *H. pylori* strains tested so far [9], [20]. Other T4SSs, such as the *cag*-T4SS (*tfs1*), *tfs3* or *tfs4,* are variably present in individual *H. pylori* strains [20]. Plasmid transfer from a donor into a recipient strain is supposed to occur via plasmid DNA release by the donor and subsequent transformation of the recipient by the *comB* system [9]. Thus, any one of the known T4SSs could be involved in conjugative plasmid transfer (Fig. 1B). In strain P12, all four T4SS are present, whereas P8 carries *comB* and *cag*-PAI only [20].

To verify conjugative transfer of plasmids pHel4 or pHel12 between *H. pylori* strains, a chloramphenicol acetyltransferase gene (*cat_GC_*) was inserted either upstream or downstream of the *mobA* gene (pHel4[*mccA::cat*]; pHel12::*cat*), which leaves the *mob* region intact (Fig. 1A). The donor strain (P8, P12) carried a *recA* deletion, whereas the recipient strain (P12) was streptomycin- (*str*) or kanamycin resistant (*moeB::aphA*-3) (Fig. 1B). Transfer of the plasmid was monitored by screening transconjugants containing the *cat_GC_* gene on the plasmid and the streptomycin or kanamycin (Str/Kan) resistance in the recipients chromosome (Fig. 1B). The *recA* mutation in the donor avoided a possible transfer of the chromosomal resistance marker from the recipient back into the donor strain by transformation. This guaranteed a transfer of plasmid pHel4 or pHel12 into the recipient (Fig. 1B). The unidirectional plasmid transfer from donor to recipient was verified by RAPD PCR (data not shown).

### Verification of Plasmid Transfer between Different *H. pylori* Strains

**Figure 2:**
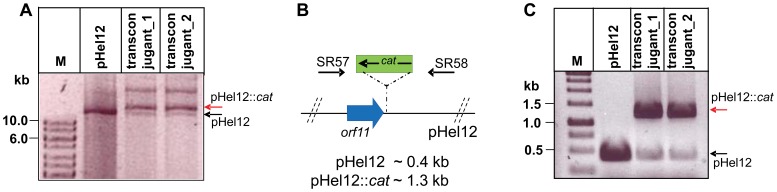
Confirmation of pHel plasmid transfer into transconjugants. (A) Isolation of plasmid DNA from transconjugants after co-cultivation of *H. pylori* P12 donor and recipient strains. Isolation of plasmid DNA after co-cultivation of *H. pylori* P12 pHel12::*cat* (donor) with *H. pylori* P12[*moeB::aphA-3*]. Presence of the donor plasmid pHel12::*cat* in the recipient strain (red arrow, lane 3,4) and of the recipient plasmid pHel12 (black arrow, lanes 3 and 4) is shown. Lane 1: 1 kb ladder, lane 2: pHel12, lane 3: transconjugant 1, lane 4: transconjugant 2. (B) Scheme explaining the PCR for the specific detection of donor- and recipient plasmid in transconjugants using oligos SR57 and SR58. (C) PCR fragments obtained with primers SR57/SR58 for the donor plasmid (red arrow, 1.3 kb) and for the original pHel12 plasmid of the recipient strain (black arrow, 0.4 kb). Lane 1, 1 kb ladder, lane 2, pHel12 used as template, lane 3 and 4, plasmid DNA from transconjugants 1 and 2 used as template.

Co-cultivation of *H. pylori* P8 or P12 as donor strains and P12 as recipient strain resulted in transfer of plasmids pHel4 or pHel12 at a frequency between 10^−5^ and 10^−6^, respectively (agar plate mating, see Materials and Methods) (Fig. 1C columns 1 and 5). The inactivation of the relaxase gene (pHel4[*mobA::cat,* pHel12[Δ*mobA*::*cat*], reduced the plasmid transfer rate slightly but not significantly (Fig. 1C, columns 3 and 7). The transferred plasmid pHel12::*cat* was successfully reisolated from the recipient strain after mating. The transferred as well as the indigenous plasmids co-existed in the recipient strain, although the transferred plasmid carrying the *cat* resistance gene was found in higher quantities, as demonstrated by gel electrophoresis and PCR analysis (Fig. 2A–C).

To further verify and expand these data to other *H. pylori* strains, co-incubation experiments were performed using *H. pylori* strain P8 as a donor with a set of different recipient strains, such as *H. pylori*, P1, P8, P12, or B128. Plasmid transfer was observed with these independent recipient strains at slightly different rates (data not shown). This suggested that independent *H. pylori* strains can act as recipients for plasmid DNA transfer.

### Plasmid Transfer Takes Place by Both, DNaseI-Sensitive Natural Transformation and a DNaseI-Resistant Transfer Mechanism

To study the mechanism of plasmid transfer, for all subsequent experiments the same strain, *H. pylori* P12, was used as donor (*recA*::*erm*) and as recipient (*str* or *moeB*::*aphA-3*) strain. This strategy ruled out potential plasmid incompatibilities or DNA restriction mechanisms [24] that might otherwise affect plasmid transfer efficiency. Furthermore, for strain P12 the complete genome sequence is available [20], so that all T4SS and relaxase genes present in the genome are known. To further corroborate the plasmid transfer as a plasmid mobilisation event, plasmid transfer was analysed with the addition of DNaseI, to remove extracellular DNA and thus to abolish natural transformation. Co-cultivation of *H. pylori* P8 or P12 carrying pHel4[*mobA*::*cat*] or pHel12[Δ*mobA*::*cat*] (donor) and P12Δ*moeB::aphA*-3 (recipient) with DNaseI treatment, revealed a significant reduction (p<0.05) in transfer rates between intact and *mobA*-defective plasmids for both P8 and P12 donor strains (Fig. 1C, compare columns 2 versus 4 and 6 versus 8). These data are best explained by natural transformation of extracellular released plasmids pHel4 or pHel12 (e.g. by lysis of bacteria), as long as no DNaseI was present (Fig. 1B, blue, dashed arrows). The addition of DNaseI destroyed extracellular plasmid DNA and abolished transformation-mediated plasmid transfer, whereas the knockout of the *mobA* relaxase gene is supposed to obstruct conjugative plasmid DNA transfer only. Thus, our data suggest that the relaxase MobA has a small but significant effect on DNaseI-resistant plasmid transfer of pHel4 and pHel12, which verifies MobA as a functional relaxase for *H. pylori* plasmids. However, since inactivation of *mobA* in the presence of DNaseI did not completely abolish plasmid DNA transfer, a further DNaseI-resistant transfer of pHel plasmids seems to occur, which is independent of the plasmid-encoded relaxase MobA.

### Conjugative DNaseI-Resistant Plasmid Transfer is Mediated by *comB*, but no Other T4SS of *H. pylori* P12

Certain cryptic plasmids of *H. pylori* carry the relaxase gene *mobA*, but none of them contain a complete T4SS mediating its conjugative transfer. We therefore asked which T4SS is responsible for the DNaseI-resistant plasmid mobilisation between *H. pylori* strains. Since up to four unique T4SSs have been described in different *H. pylori* isolates, one (or several) of the chromosomally encoded T4SSs (*tfs1-tfs4*) might mediate plasmid mobilisation.

**Figure 3:**
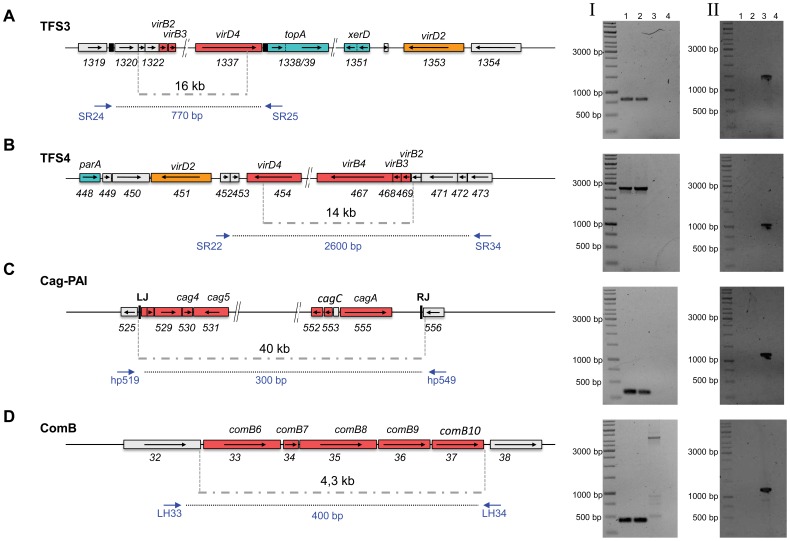
Deletion of individual T4SS from the genome of *H. pylori* strain P12 by the marker-free counterselection procedure. (A) Schematic representation of Tfs3 and verification of its deletion. For deletion of the complete Tfs3 (16 kb region), the flanking sequences were amplified using oligos SR17/SR18 and SR30/SR35 (see Table S1 for primers) and cloned into the pBluescript vector either with or without the *rpsL-erm* cassette in-between. Verification of the correct deletion was obtained by DNA amplification of a genomic fragment using oligos SR24/SR25 resulting in a PCR fragment of 0.77 kb, as expected (gel I). The PCR fragment was further verified by DNA sequencing. Furthermore, amplification of an internal region of Tfs3 (primers WS542/355) resulted in the expected PCR product from wt, but not P12ΔT4SS chromosomal DNA (gel II) (B) For deletion of the complete Tfs4 (14 kb region) the flanking sequences were amplified using oligos SR13/SR14 and SR32/SR34 (see Table S1 for primers) and cloned into the pBluescript vector either with or without the *rpsL-erm* cassette enclosed. Verification of the correct deletion was obtained by DNA amplification of a genomic fragment using oligos SR22/SR34, resulting in a 2.6 kb fragment, as expected (gel I). The PCR fragment was further verified by DNA sequencing. Amplification of an internal region of Tfs4 (primers SR48/49) resulted in the expected PCR product from wt, but not P12ΔT4SS chromosomal DNA (gel II) (C) The complete *cag*-PAI was deleted by the *rpsL*-counterselection procedure. Flanking fragments were cloned using oligos JP22/23 and JP24/25 for PCR (see Table S1 for primers). The correct deletion was verified by the generation of a PCR fragment of 300 bp spanning the deletion, as generated by primers hp519 and hp549. The PCR fragment was further verified by DNA sequencing. Amplification of an internal region of the *cag*-PAI (primers WS418/HP542f) resulted in the expected PCR product from wt, but not P12ΔT4SS chromosomal DNA (gel II). (D) Deletion of the *comB6*– *comB10* genes of the *comB* T4SS by the *rpsL*-counterselection procedure (4.3 kb). Flanking regions were cloned using oligos AK59/AK65 and DHO10/DHO11 for PCR (see Table S1 for primers). The deletion was verified by the generation of a 400 bp fragment, whereas the intact *comB* locus resulted in a 4300 bp fragment. Amplification of an internal region of comB (primers DHO14/DHO15) resulted in the expected PCR product from wt, but not P12ΔT4SS chromosomal DNA (gel II). Components with sequence homology to the VirB/D4-System of *A. tumefaciens* are shown in red; other elements important for the potential function of the T4SS are depicted in blue or orange. Lane M, 1 kb ladder; lane 1 donor strain P12ΔT4SS; lane 2, recipient strain P12ΔT4SS; lane 3, P12 wt strain; lane 4: water control.

**Figure 4:**
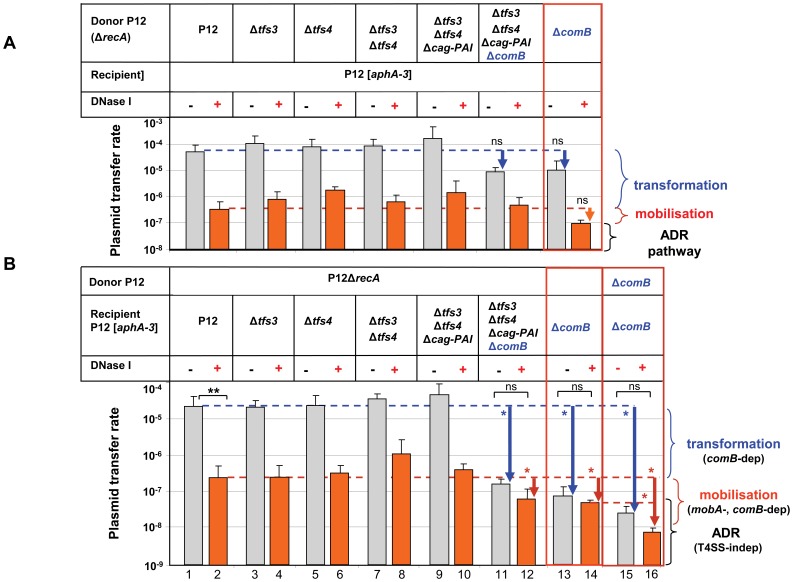
Only the *comB* but no other T4SS in the donor and recipient is involved in conjugative plasmid DNA transfer between *H. pylori* strains. (A) DNA transfer experiments of plasmid pHel12::*cat* were performed from a P12Δ*recA* donor strain carrying complete marker-free deletions (*rpsL-erm* counter-selection procedure) of one or several T4SS into a wild-type P12 recipient strain in the absence (−) or presence (+) of DNaseI. The donor strain carries a *recA* deletion to render it non-transformable. As recipient strains, a P12 wild-type strain was used carrying a Δ*moeB*(HPP12_765)::*aphA*-*3* insertion conferring kanamycin resistance. Growth of chloramphenicol/kanamycin double-resistant clones indicated a unidirectional transfer of plasmid pHel12::*cat* to the recipient strains. (B) DNA transfer experiments of plasmid pHel12::*cat* from a wt P12Δ*recA* donor into different P12 recipient strains carrying precise marker-free deletions of one or several T4SSs. Transfer rates were determined as the number of transconjugants/cfu/ml. Data shown are mean values of at least three independent experiments including standard deviations. Blue and red vertical arrows indicate the reduction in transformation and DNaseI-resistant plasmid DNA transfer rates, respectively. Data shown are mean values of at least three independent experiments including standard deviations. *, p<0.05; **, p<0.01, ns, not significant (p>0.05) according to students t-test. P12Δ*tfs3*/Δ*tfs4*/Δc*ag-PAI*/Δ*comB* is also refered to as P12ΔT4SS.

To study the role of these T4SSs for plasmid transfer, all T4SSs were successively removed from the genome by a marker-free genetic deletion method, the *rpsL* counterselection procedure [25]. First, the complete *tfs3* and *tfs4* systems were deleted in the P12 donor strain and the correct deletions were verified by PCR analysis (Fig. 3) (see Materials and Methods and Tables 1, 2 and Table S1 for details of plasmid construction). Removal of TFS3 or TFS4 in the donor (Fig. 4A) or in the recipient strain (Fig. 4B) did not significantly change the plasmid transfer frequency, neither without nor with DNaseI. Also a double mutant (P12Δ*tfs3*Δ*tfs4*) did not show a defect in plasmid transfer. In the next step the *cag*-PAI was removed by the same procedure (Fig. 3C), resulting in a triple mutant (P12Δ*tfs3*Δ*tfs4*Δ*cag*-PAI). Again, plasmid transfer rates were not reduced, as compared to the P12 wt situation (Fig. 4A, B). An additional deletion of the *comB* system in the donor strain (P12Δ*tfs3*Δ*tfs4*Δ*cag*-PAIΔ*comB*, also termed P12ΔT4SS), or deletion of the complete *comB* system only, did not result in a significant change of plasmid transfer rate (Fig. 4A, columns 11 and 12). However, the same deletions in the recipient strain significantly reduced plasmid transfer rates, especially in experiments without DNaseI (transformation) but also to a lower extent with DNaseI (p<0.05) (Fig. 4B, columns 11 and 12). Knockout of the *comB* system only in the recipient strain gave very similar results as the quadruple mutants (Fig. 4B, columns 13 and 14). A simultaneous deletion of the *comB* T4SS in both, the donor as well as the recipient strain, maximally reduced plasmid transfer rates, supporting the role of the donor strain *comB* T4SS for DNaseI-resistant plasmid transfer (Fig. 4B, columns 15 and 16). To exclude any insufficient DNA degradation by DNaseI and therefore residual transformation, the extracellular DNA of the co-cultivation assay with and without DNaseI was extracted, sterile-filtrated and used for transformation of the *H. pylori* P12 wild type (wt) strain. The non-treated, but not the DNaseI-treated extract resulted in transformation of P12, indicating that the DNase used was efficient during the co-incubation conditions (data not shown). Plating of the donor and recipient strains separately (without co-incubation) on double selective media did not result in any resistant bacteria, which excluded the generation of any spontaneous mutants (data not shown).

**Table 1:** Bacterial strains used in this study.

Strain	Plasmid	Genotype and reference
*DH5*α		*Eschericha coli K12*
ATCC43526		*H. pylori* reference strain, ATCC
*Hp* P1		*H. pylori* clinical isolate (69A) University Hospital Amsterdam, The Netherlands
*Hp* P1		*moeB::aphA3*
*Hp* P8		*H. pylori* clinical isolate (196A) University Hospital Amsterdam, The Netherlands
*Hp* P8	pHel4 [*mccC::cat*]	*recA::erm*
*Hp* P8	pHel4 [*mobA::cat*]	*recA::erm*
*Hp* P12	pHel12	*H. pylori* clinical isolate (888-0) [39]
*Hp* P12	pHel12	*rpsL* (str)
*Hp* P12	ΔpHel12	*recA::erm*
*Hp* P12	pHel12*::cat*	*recA::erm*
*Hp* P12	pHel12 [*mobA::cat*]	*recA::erm*
*Hp* P12	pHel12*::cat*	*recA::erm;* Δ*tfs3*
*Hp* P12	pHel12*::cat*	*recA::erm;* Δ*tfs4*
*Hp* P12	pHel12*::cat*	*recA::erm;* Δ*comB*
*Hp* P12	pHel12*::cat*	*recA::erm;* Δ*tfs3;* ΔvirD2_tfs3_
*Hp* P12	pHel12*::cat*	*recA::erm;* Δ*tfs4;* ΔvirD2_tfs4_
*Hp* P12	pHel12*::cat*	*recA::erm;* Δ*tfs3*; Δ*tfs4*
*Hp* P12	pHel12*::cat*	*recA::erm;* Δ*tfs3;* Δ*tfs4;* ΔvirD2_tfs3_; ΔvirD2_tfs4_
*Hp* P12	pHel12*::cat*	*recA::erm;* Δ*tfs3*; Δ*tfs4*; Δ*cagPAI*
*Hp* P12	pHel12*::cat*	*recA::erm;* Δ*tfs3*; Δ*tfs4*; Δ*cagPAI;* Δ*comB*
*Hp* P12	pHel12*::cat*	Δ*tfs3*; Δ*tfs4*; Δ*cagPAI;* Δ*comB ::rpsl-erm*
*Hp* P12	pHel12 [*mobA::cat*]	Δ*tfs3*; Δ*tfs4*; Δ*cagPAI;* Δ*comB::rpsl-erm*
*Hp* P12	pHel12	*moeB::aphA3*
*Hp* P12	pHel12	*moeB::aphA3;* Δ*tfs3*
*Hp* P12	pHel12	*moeB::aphA3;* Δ*tfs4*
*Hp* P12	pHel12	*moeB::aphA3;* Δ*comB*
*Hp* P12	pHel12	*moeB::aphA3;* Δ*tfs3*, Δ*tfs4*
*Hp* P12	pHel12	*moeB::aphA3;* Δ*tfs3*, Δ*tfs4*, Δ*cagPAI*
*Hp* P12	pHel12	*moeB::aphA3;* Δ*tfs3*; Δ*tfs4*; Δ*cagPAI;* Δ*comB*

**Table 2:** Plasmids used in this study.

Plasmid	Characteristics	Reference
**pAK23**	pMin1, upstream region of *comB6, downstream region of comB10,*sepa-rated by *aphA-3* cassette, Tet^R^, Erm^R^	[10]
**pBluescript II SK+**	*ori*_colE1_, *ori*_f1(+)_, *lacZ*, M13 forward−/reverse primer binding sites, Amp^R^	Stratagene
**pCR2.1-TOPO**	*ori*_colE1_, *ori*_f1_, *lacZ*, M13 forward-, reverse- and T7 promotor/primer binding sites,Amp^R^, Kan^R^	Invitrogen
**pDH29**	pBluescript II KS^+^ *recA*::*erm*, Amp^R^, Erm^R^	[40]
**pJP44 (** ***rpsL-erm*** **)**	Δ*cagPAI::rpsL-erm*	This study
**pSR11**	pBluescript IISK *rpsL-erm*-cassette in *BamH*I	This study
**pSR12**	Deletion of T4SS *tfs3*; *rpsL-erm*	This study
**pSR13**	Deletion of T4SS *tfs3 rpsL-erm* deleted	This study
**pSR14**	Deletion of T4SS *tfs4*; *rpsL-erm*	This study
**pSR15**	Deletion of T4SS *tfs4; rpsL-erm deleted*	This study
**pSR18**	Deletion of T4SS of *cagPAI; rpsL-erm* (pJP44+*rpsL-erm*)	This study
**pSR19**	Deletion of T4SS *comB; rpsL-erm* (pAK23+*rpsL-erm*)	This study
**pSR20**	pSP76 carrying *aphA-3* cassette; in *moeB* locus	This study
**pSR21**	Deletion of *virB4/topA*; Insertion of *rpsL-erm* cassette	This study
**pSR23**	Insertion of *virB4/topA* [ATCC*43526*]	This study
**pSR24**	Deletion of *orf12GH* in pHel12; Insertion of *aphA-3*-cassette	This study
**pSR25**	Deletion of *orf12GH* in pHel12; Insertion of *cat_GC_-*cassette	This study
**pSR26**	Deletion of *orf12C* in pHel12; Insertion of *cat_GC_* cassette	This study
**pSR27**	Deletion of *orf12C* in pHel12; Insertion of *aphA-3-*cassette	This study
**pSR28**	Insertion of *cat_GC_* cassette between *orf4M* and *orf4A* in pHel12	This study
**pSR29**	Deletion of *tfs3* including chromosomal relaxase using *rpsL-erm*	This study
**pSR33**	Deletion of *comE3*; Insertion of *aphA-3*-cassette	This study
**pWS48**	pBluescript II KS^+^ carrying *recA* (partial)::*cat*, Amp^R^, Cam^R^	[40]
**pWS102**	Deletion of *vacA*; Insertion of *aphA-3*-cassette	This study

In conclusion, these data suggest that none except the *com*B T4SS contributes to plasmid transfer by transformation as well as a DNaseI-resistant mobilisation.

### Plasmid-Encoded Relaxase MobA, but not Chromosomally Encoded Relaxases Contribute to Plasmid Transfer

**Figure 5:**
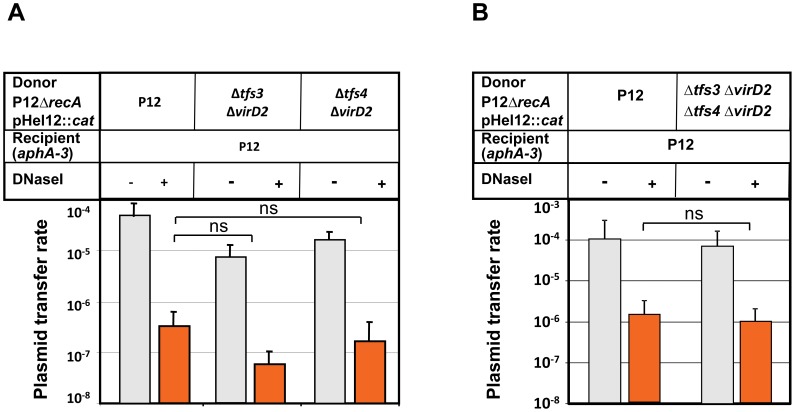
Role of chromosomally encoded relaxases for transfer of plasmid pHel12. (A) DNA transfer rates of plasmid pHel12::*cat* from a P12Δ*recA* donor carrying precise marker-free deletions of *tfs3* or *tfs4* including the adjacent relaxase genes *virD2* (see Materials and Methods for construction of the deletions) into a P12 recipient strain carrying a chromosomal kanamycin resistance gene (*aphA*-3). (B) DNA transfer of plasmid pHel12::*cat* from a P12Δ*recA* donor carrying marker-free deletions of both, *tfs3* and *tfs4* including the adjacent relaxase genes *virD2* into a P12 recipient strain. Transfer rates were determined as the number of transconjugants/cfu/donor. Data shown are mean values of at least three independent experiments including standard deviations. *, p<0.05; ns, not significant (p>0.05) according to students t-test.

The plasmid-encoded relaxase MobA contributed to DNaseI-resistant plasmid transfer of pHel4 as well as pHel12 (Fig. 1C). *H. pylori* P12 harbours two chromosomally-encoded VirD2-homologous relaxases, HPP12_1353 and HPP12_0451, located next to *tfs3* and *tfs4*, respectively (Fig. 3A, B). Therefore, it could not be excluded that these putative chromosomal relaxases partially compensate for the deletion of the plasmid-encoded relaxase in strain P12. We therefore sought to analyse the effect of the plasmid- as well as chromosomally encoded relaxases in strain P12 on plasmid transfer. The original deletions of TFS3 or TFS4 described above left the VirD2-homologous relaxases in the chromosome. Therefore, the complete *tfs3* and *tfs4* systems, including the adjacent relaxase genes were deleted using the *rpsL* counter-selection procedure. Precise deletions were verified by PCR as described for the *tfs3/4* deletions (data not shown, see Material and Methods section and Table S1 for primers) Removal of HPP12_1353 together with *tfs3* (Δ*tfs3*Δ*virD2*), as well as HPP12_0451 with *tfs4* (Δ*tfs4*Δ*virD2*) did not significantly change the plasmid transfer rates (Fig. 5A). Also a double mutant (Δ*tfs3*Δ*virD2*, Δ*tfs4*Δ*virD2*) did not have a significant effect on plasmid transfer, as compared to the transfer rates between P12 strains (Fig. 5B). In conclusion, the chromosomally-encoded relaxases HPP12_1353 or HPP12_0451, located adjacent to TFS3 or TFS4, respectively, were not involved in plasmid DNA transfer, neither in a DNaseI-sensitive nor a DNaseI-resistant manner.

### A T4SS-Independent Alternate DNaseI-Resistant (ADR) Mechanism of Plasmid Transfer in *H. pylori*

**Figure 6:**
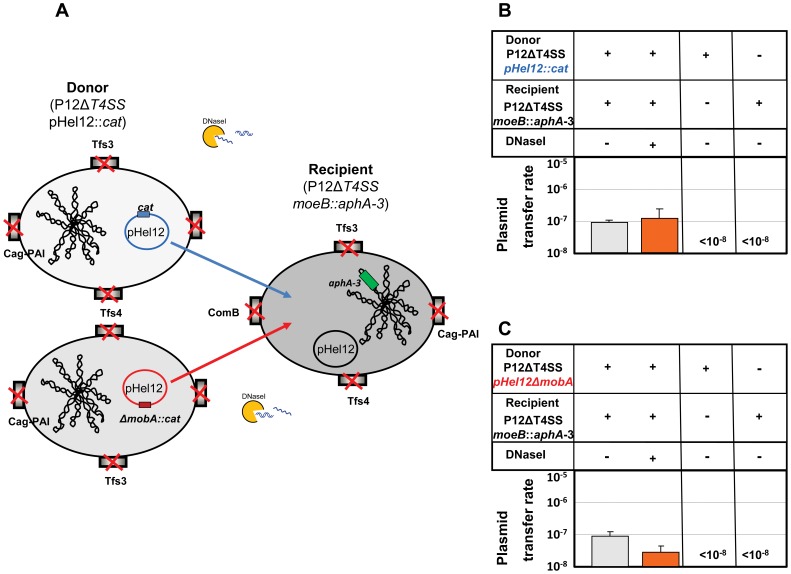
Marker-free deletions of all T4SS in the *H. pylori* P12 (P12ΔT4SS) donor and recipient strain prove the existence of an alternate DNaseI-resistant (ADR) pathway of DNA transfer. (A) Schematic depiction of DNA exchange between *H. pylori* P12 donor and recipient strains both of which are devoid of any T4SS to support natural transformation or conjugative plasmid transfer. The recipient strain carries a kanamycin resistance gene (*aphA-3*) in the chromosomal *moeB* locus, the donor strain either carries a *cat* gene in pHel12 (pHel12::*cat*) (B), or *cat* replaces the *mobA* gene (pHel12Δ*mobA*::*cat)* (C). DNA transfer rates of plasmid pHel12::*cat* (B) or pHel12Δ*mobA*::*cat* (C) from a P12ΔT4SS donor strain into a P12ΔT4SS recipient strain carrying an *aphA-3* cassette in the *moeB* locus are shown. Transfer rates were determined as the number of transconjugants/cfu/ml. Data shown are mean values of at least three independent experiments including standard deviations.

Our data provided evidence that plasmids can be transferred between *H. pylori* strains by natural transformation, as well as by conjugation. Both routes of DNA transfer are strictly dependent on the *comB* T4SS. For conjugation or mobilisation of plasmids between *H. pylori,* the *mobA* relaxase is essential. However, in our experiments DNaseI-resistant plasmid transfer was clearly seen in strains without any T4SS (P12ΔT4SS) used either as donor or as recipient (Fig. 4). To unequivocally prove the existence of such an alternate DNA transfer pathway in *H. pylori* operating independently of any T4SS, an experiment was designed using a P12ΔT4SS strain as a donor as well as a recipient. Such a strain cannot be transformed by plasmid DNA [9], nor can it act as a donor or recipient for conventional conjugative DNA transfer, since it is does not contain any T4SS (Fig. 6A).

Co-incubation experiments using such a pair of strains showed that plasmid pHel12::*cat* can be transferred between *H. pylori*, albeit at a lower efficiency as that seen for natural transformation or mobilization (∼1×10^7^), but still at a reasonable rate (Fig. 6B). Furthermore, the additional deletion of the *mobA* gene of pHel12 (*pHel12ΔmobA::cat*) did not abrogate the transfer between the P12ΔT4SS strains. However, control plating experiments with the donor or the recipient strain alone on double selective media did not result in any resistant bacteria, excluding spontaneous mutations of the donor or the recipient strain as a source for the double resistant bacteria. Without any T4SS extracellular plasmid DNA should not contribute to this type of plasmid transfer via transformation, which is supported by the fact that a similar low rates of DNA transfer were seen with and without addition of DNaseI (Fig. 6B, C). Finally, the plasmids from the double resistant bacteria were isolated and confirmed to be pHel12::*cat*. The recipient strain carrying the transferred plasmid could also be verified as P12ΔT4SS_*moeB::aphA*-*3*.

In conclusion, these experiments clearly show that an alternative pathway for plasmid exchange is existent in *H. pylori*, which is distinct from natural transformation and conventional conjugation. We suggest naming this pathway as ADR pathway.

## Discussion

The existence of plasmids in *H. pylori* has been known for more than twenty years [12]. More recently, a subgroup of *H. pylori* plasmids has been described that carries putative mobilisation genes [13], [17], [20], [26], which might be involved in conjugative transfer between different *H. pylori* strains. This is of particular interest, since such plasmids might be involved in the generation of genetic variation in *H. pylori* [13]. Here we studied the transfer of such plasmids between identical and different, unrelated *H. pylori* strains. Since *H. pylori* is naturally competent for DNA transformation, the analysis of conjugative transfer of plasmid DNA between *H. pylori* is more complicated than in bacteria without natural transformation competence, since both processes are superimposed (Fig. 1B). A simple transfer of the chromosomal resistance gene marker (*str, aphA-3*) from the recipient bacteria into the donor strain by natural transformation, which would result in double resistant pseudo-transconjugants should be avoided. We therefore generated a *recA* mutation in the donor strains making them unable to integrate chromosomal markers (Fig. 1B).

Generally, plasmid mobilisation is defined by the DNaseI-resistant transfer of DNA and its dependence on a functional T4SS, mediating the conjugative transfer. Classical conjugative transfer is also dependent on a functional relaxase, which nicks one strand of the plasmid DNA at a specific sequence in the *mob* site. Rolling circle replication elongates the DNA strand and the relaxase bound to the 5′-end of the single-stranded DNA then mediates the specific transfer of the nucleoprotein complex into the T4SS and the recipient cell [27].

We show here for the first time that the *comB* T4SS, which up to now has been associated with natural transformation competence only, can accomplish DNaseI-resistant plasmid DNA transfer as well. We identified two distinct mechanisms of DNaseI-resistant plasmid transfer, one mechanism being dependent on a functional plasmid-encoded relaxase (plasmid mobilisation) and one transfer mechanism completely independent of any relaxase, and even any T4SS, but nevertheless resistant to DNaseI (ADR). Conjugative transfer of *H. pylori* shuttle vectors carrying an RP4 *oriT* sequence has been reported to occur between different *H. pylori* isolates [28]. These plasmids did not carry a relaxase gene on the plasmid, but contained an origin of transfer (*oriT*) from the broad host range plasmid RP4. Conjugative transfer of these plasmids was reported to be dependent on a chromosomal relaxase gene (*rlx1*, *hp0996*) and the coupling protein HP1006 [28]. Plasmid transfer with the endogenous plasmid pHel12, which carries a *H. pylori*-specific relaxase gene and no RP4 *oriT,* has been shown here to be independent of chromosomal relaxases.

The *comB* T4SS is well established as being absolutely necessary for transformation-mediated DNA uptake into *H. pylori* [9]. Our data show that in addition to transformation, *comB* is also important for DNaseI-resistant plasmid DNA transfer between *H. pylori* strains, generally designated as conjugative transfer or mobilisation. First, a *comB* deletion in the recipient strain significantly reduced plasmid DNA transfer rates, both for transformation and DNaseI-resistant transfer (Fig. 4B, columns 11–16). Second, a *mobA* deletion in pHel12 resulted in a significant reduction of DNA transfer (Fig. 1C), whereas other T4SS did not have any effect. Thus, we showed here for the first time that a *comB*-dependent mobilisation of pHel12 plasmid is an obvious pathway of plasmid transfer between *H. pylori*.

Surprisingly, a P12 recipient strain without any T4SS (P12ΔT4SS), for which DNA transformation was completely abolished, did not exhibit a significantly different efficiency of DNaseI-sensitive or resistant pHel12 plasmid transfer rate under contact-dependent co-incubation conditions (Fig. 4B, columns 11/12) than a P12Δ*comB* strain (Fig. 4B, columns 13/14). Definite proof for pHel12 plasmid transfer in the absence of any T4SS allowing natural transformation or conjugation was coming from a co-incubation experiment with donor and recipient strains devoid of any T4SS (P12ΔT4SS). Since plasmid transfer could be demonstrated under these conditions, our data prove that plasmid transfer between *H. pylori* can occur by a novel, hitherto not characterized T4SS-independent, but DNaseI-resistant pathway. We propose to designate this pathway as alternate DNaseI-Resistant pathway (ADR pathway). The mechanism for plasmid DNA transfer via the ADR pathway is unclear and currently investigated in detail in our lab. From the current literature we would envisage two possible mechanisms for plasmid DNA transfer in *H. pylori* via the ADR pathway, (i) either outer membrane vesicles (OMVs) [29] or (ii) nanotubes [30]. Nanotubes have been recently described as variously sized tubular extensions connecting Gram-positive or Gram-negative bacteria, allowing non-conjugative DNaseI-resistant plasmid transfer between the same or even different bacterial species. The production of OMVs has been described for *H. pylori*, especially for a potential delivery of proteins (e.g. VacA) or peptidoglycan into host cells [31], [32], but not for transfer of plasmid DNA.

Taken together, these data demonstrate that three different mechanisms of plasmid DNA exchange are operating in *H. pylori*. Whether these different mechanisms are active at the same time, or whether there is a spatial or temporal control for one or the other mechanism, is currently not known. However, it is possible that by knockout of the *comB* system, e.g. in the donor strain, the ADR pathway may compensate for the defect in plasmid transfer, which might explain the small effect of a P12Δ*comB* donor strain on plasmid DNA transfer efficiency (Fig. 4A). Furthermore, our data were exclusively generated under *in vitro* conditions. The *in vitro* plasmid transfer rates cannot easily be transferred to the situation of the bacteria under *in vivo* conditions in the stomach mucosa. It is well possible that one or the other pathway might be turned on or off under *in vivo* conditions. In a recombination-based *in vivo* expression technology (RIVET) screen, a promoter in the mG27 *H. pylori* strain was identified, which turned on the expression of the plasmid-encoded *mobA* gene under *in vivo* conditions in the mouse stomach [33], indicating that plasmid transfer might be enhanced when the bacteria are in their natural environment, the stomach mucosa. In the *in vitro* situation, natural transformation seems to represent the most efficient way of plasmid transfer, followed by conjugative transfer, whereas the ADR pathway contributes only minimally. The relative contribution of one or the other pathway under *in vivo* conditions has to be determined in future.

## Materials and Methods

### Bacterial Strains and Growth Conditions

In this study *H. pylori* strains P8 (originally isolated as 196A) and P12 (originally isolated as 888-0) [34] were used. For routine culture, *H. pylori* strains were grown on GC agar plates (Oxoid) supplemented with horse serum (8%), vancomycin (10 µg/ml), trimethoprim (5 µg/ml), and nystatin (1 µg/ml) (serum plates). Erythromycin (10 µg/ml), chloramphenicol (6 µg/ml), kanamycin (8 µg/ml), and streptomycin (250 µg/ml) were added to select for transformants or screen colonies for resistance to either drug. Inoculated plates were incubated for 24 to 48 h under microaerobic conditions (85% N_2_, 10% CO_2_, 5% O_2_) at 37°C. *Escherichia coli* DH5α was grown on Luria-Bertani (LB) agar plates or in LB liquid medium [35] supplemented with ampicillin (100 µg/ml), chloramphenicol (30 µg/ml), erythromycin (250 µg/ml), or kanamycin (40 µg/ml), as appropriate.

### Electroporation, Natural Transformation and Conjugation of *H. pylori*

Transformation of *H. pylori* strains was performed with plasmid or chromosomal DNA as described earlier [34]. Bacteria were harvested from serum plates and suspended to an optical density at 550 nm (OD_550_) of 0.2 in Brucella broth (BB) containing 10% fetal calf serum. DNA was added (1 µg), and incubation was extended for 4 h under microaerophilic conditions before the suspension was plated on selective serum plates. For electroporation of *H. pylori*, bacterial cells were harvested from serum plates and suspended in 1 ml of phosphate-buffered saline (PBS) solution. For each electroporation, bacteria were diluted to an OD_550_ of 1 and 1 ml bacterial suspension was washed twice with PBS and suspended first in 500 µl and then in 40 µl electroporation buffer [36]. Forty microliters of *H. pylori* competent cells was mixed with 1 to 2 µl DNA in pre-chilled 0.2 cm electroporation cuvettes. Electroporation was performed at 2.5 kV, 200 Ω, and 25 µF by a Gene Pulser (Bio-Rad, Munich, Germany). After electroporation, 1 ml of BB containing 10% fetal calf serum was added immediately to each sample. The aliquots were incubated for 4 h in a CO_2_ incubator before being plated on selective agar plates.

### DNA Manipulations and Plasmid and Strain Constructions

Cloning and DNA analysis procedures were performed according to Sambrook *et al.* [35]. Chromosomal DNA from *H. pylori* was isolated with the QIAamp tissue kit (QIAGEN, Hilden, Germany). Plasmid DNA was purified from *E. coli* using the QIAprep Spin Miniprep kit (QIAGEN, Hilden, Germany).

Deletion and replacement of genes or genomic regions were achieved using the streptomycin susceptibility counterselection strategy [25]. A *rpsL*-*erm* cassette cloned into the *Bam*HI restriction site of pBluescript IISK+ was used for all cloning procedures. For the deletion of the TFS3 system, the corresponding upstream and downstream regions were amplified by PCR using primers SR17/SR18, and SR30/SR35, respectively (Table S1). Likewise, *tfs4* upstream and downstream regions were amplified using primers SR13/SR14 and SR32/SR34, respectively. Upstream and downstream regions were cleaved with *Xho*I/*Cla*I and *Not*I/*Sac*II, respectively, and cloned with or without the *rpsL*-*erm* cassette into the corresponding sites of pBluescript IISK+. The plasmids obtained were used in sequential transformations to generate marker-free deletions of the corresponding T4SS.

To inactivate the ComB system, *comB6-comB10* were deleted using the *rpsL-erm* system and plasmid pAK23 [10]. For deletion of the *cag*-PAI, a plasmid containing the left and right flanking gene regions of the *cag*-PAI [37], was modified by insertion the *rpsL-erm* cassette. To monitor plasmid transfer between *H. pylori* strains, a chloramphenicol resistance cassette (*cat_GC_*) was inserted between *orf4M* and *orf4A* in pHel12 using an inverse PCR reaction with primers SR53 and SR54. The *cat_GC_* cassette (pWS48) was cloned via a *Bam*HI restriction site. Homologous recombination in plasmid pHel12 resulted in pHel12*::cat_GC_*.

For deletion of the plasmid-encoded relaxase of pHel12, a plasmid from a shotgun library was used as template and after inverse PCR using primers SR41/SR52, the *cat_GC_* cassette was inserted via a *Bam*HI restriction site. In pHel4 the *cat_GC_* cassette was inserted within *orf4C* to disrupt the corresponding gene. To delete the chromosomal relaxases within *tfs3* and *tfs4*, plasmids pSR29 and pSR31 were generated using primer pairs SR17/SR18 and SR69/SR70 (*tfs3*/Rel deletion), or SR71/SR72 and SR28/SR34 (*tfs4*/Rel deletion). As already described above, the *rpsL-erm* counterselection strategy was applied to generate marker-free *H. pylori* mutant strains.

### Mating Experiments

To monitor transfer of the donor plasmid into the recipient strain, donor strains were provided with a *cat_GC_* cassette in the plasmid. For selection of the recipient strain, either an *aphA-3* cassette was inserted into the recipients chromosomal *moeB* locus, using plasmid pSP76-*aphA-3*, or a streptomycin resistant P12 mutant (pEG21) was used. To prevent transformation of donor strain with chromosomal DNA from recipient strain, the *recA* gene was deleted in the *H. pylori* donor strains by transformation with the plasmid pDH29 (*recA::erm*). Donor and recipient strains were harvested after 24 h of growth on selective GC agar plates and suspended in 1 ml of BB. For mating experiments between *H. pylori* 1.5·10^7^ cells were used. 25 µl donor and 25 µl recipient cell suspension were either mixed with 25 µl DNaseI (Roche; 1 mg/ml in 20 mM Tris-Cl (pH 7.5), 1 mM MgCl_2_) each in separate tubes and pre-incubated for 30 min at 37°C and 10% CO_2_, or left untreated. Subsequently donor and recipient cells were mixed, collected at 1000 *g* for 5 min and resuspended either in 50 µl DNaseI or 50 µl BB. The cells were spotted onto non selective GC agar plates directly and incubated over night at 37°C under microaerobic conditions. After incubation the cells were harvested and resuspended in 1 ml BB. The cell suspension was serially diluted and 100 µl were plated on GC agar plates containing chloramphenicol to determine the CFU/ml of the donor strain and 100 µl and harvested ∼800 µl cell suspension were plated on appropriate double selective GC agar plates (chloramphenicol and kanamycin or streptomycin) to determine the number of transconjugants. The plasmid transfer rates were determined by colony counting and are presented as numbers of transconjugants per donor cfu. Plasmid sequence accession numbers: pHel4: NC_004950; pHel12: CP001218.

### PCR-based RAPD Fingerprinting

To differentiate between donor (P8) and recipient (P12) *H. pylori* strain in mating experiments a RAPD DNA fingerprinting method was applied [38]. PCR was carried out in 25 µl containing 20 ng of *H. pylori* genomic DNA, 3 mM MgCl_2_, 20 pmoles of primer, 1 U of Takara® Taq DNA polymerase, 250 µM each of dCTP, dGTP, dATP and dTTP. The cycling program was four cycles of [94°C, 5 min; 40°C, 5 min; and 72°C, 5 min; low stringency amplification], 30 cycles of [94°C, 1 min; 55°C, 1 min; and 72°C, 2 min; high stringency amplification], and a final incubation at 72°C for 10 min.

### Statistical Analysis

The values shown are means ± SD from at least three independent experiments. Students t-test was used to analyze the data. P values are indicated in the figures and were considered significant if they were <0.05.

## Supporting Information

Table S1 **Oligonucleotide Primers used in this study.** Different types of oligonucleotide primers are listed which were used for amplification of genes for cloning purposes, for verification of chromosomal deletions (e.g. complete T4S systems) or for sequencing. The cloning procedures are described in the methods section.
(DOCX)
