# Supplementary material for: Multiple Pathways of Plasmid DNA Transfer in Helicobacter pylori
Source: PLoS One. 2012 Sep 20;7(9):e45623. doi: 10.1371/journal.pone.0045623 (PMC3447787; doi:10.1371/journal.pone.0045623)
Supplement: Table S1 — Oligonucleotide Primers used in this study. Different types of oligonucleotide primers are listed which were used for amplification of genes for cloning purposes, for verification of chromosomal deletions (e.g. complete T4S systems) or for sequencing. The cloning procedures are described in the methods section. (DOCX) [file pone.0045623.s001.docx]

**Supporting Table 1: Primers used in this study**

| **Name** | **Specificity** | **Direction** | **Sequence (5`-> 3`)** | **Purpose of use or reference** |
| --- | --- | --- | --- | --- |
| **AK59** | *comB* |  | gga att cga aga aca tca taa gcg ttt | verification of deletion of *comB*-system |
| **AK65** | *comB* |  | cag aat tcc aat act tca acg gac ttt | deletion of *comB6-10* |
| **D9355** |  |  | ccg gat ccg tga tgc ggt gcg | RAPD-PCR primer, Akopyanz et al. 1992 |
| **DHO10** | *comB* |  | ggg gta ccc tta aaa atg ccc aat taa a | downstream *comB10* |
| **DHO11** | *comB* |  | cca tcg atc aaa tga tcg ctt ggc gt | downstream *comB10* |
| **DHO14** |  |  | ccg ctc gag gtc gac tca ctt caa aaa ctc agc | amplification  *comB* internal |
| **DHO15** | *comB* |  | gaa gat ctc aaa aag ggt aaa gat g | verification of deletion of *comB*-system |
| **HH64** | *rpsL-erm* |  | cag c ggatcc at gct tta taa cta tgg att | amplification of *rpsL-erm* cassette (*Bam*HI) |
| **HH66** | *rpsL-erm* |  | gc ggatcc t tac tta tta aat aat tta tag | amplification of *rpsL-erm* cassette (*Bam*HI) |
| **HP519** | *cag*-PAI |  | gct tgc ttg tat tgg cct tg | sequence flanking *cag*-PAI |
| **HP549** | *cag*-PAI |  | gca tgc aca ttc cct aaa gtg | Sequence flanking *cag*-PAI |
| **JP22** | *cag*-PAI |  | ggg gta cct tac ctt acc ggc ttt att aat g | *cag*-PAI deletion |
| **JP23** | *cag*-PAI |  | gga gat cta agg atc tga cat gtt ta | *cag*-PAI deletion |
| **JP24** | *cag*-PAI |  | gaa gat cta tcg att att tta tta gcg tta c | *cag*-PAI deletion |
| **JP25** | *cag*-PAI |  | acc gct cga gct gca gcc caa gaa ttc aaa cga c | *cag*-PAI deletion |
| **LH33** | *comB6-10* | sense | taa gag aag cgt taa gcg cg | verification deletion *comB6-10* |
| **LH34** | *comB6-10* | antisense | gag cgt ttc atc tac taa gg | verification deletion *comB6-10* |
| **SR4** | *orf4C, orf04* | sense | act gat tac ata ttc gta ata ata ttt | sequencing of deletion of *orf4C* or *orf04* |
| **SR5** | *orf4C, orf04* | antisense | aga aat cag caa tca att aga aca ac | sequencing of deletion of *orf4C* or *orf04* |
| **SR13** | Tfs4 | sense | gat cct cga gct tct aaa tct ttg cta aat | deletion of Tfs4 (XhoI) |
| **SR14** | Tfs4 | antisense | gat cat cga taa gcc caa tga aga agt gt | deletion of Tfs4 (ClaI) |
| **SR17** | Tfs3 | sense | gat cct cga gct aaa ctc tgg cgt aaa att | deletion of Tfs3 (XhoI) |
| **SR18** | Tfs3 | antisense | gat cat cga tgt ccc ttt cta tga gac tta g | deletion of Tfs3 (ClaI) |
| **SR22** | Tfs4 | sense | aca cca agt ttg tta aat ac | sequencing deletion *tfs4* |
| **SR24** | Tfs3 | sense | gag cgt ctt tga aat gcg tt | sequencing deletion *tfs3* |
| **SR25** | Tfs3 | antisense | tct cta tca gtg tca gtg gc | sequencing deletion *tfs3* |
| **SR28** | Tfs4 | sense | gat cgc ggc cgc cat tct tat tcc ttt atg tt | deletion of *tfs4* (NotI) |
| **SR30** | Tfs3 | sense | gat cgc ggc cgc aca caa agc acc caa agt ta | deletion of *tfs3* (NotI) |
| **SR32** | Tfs4 | sense | gat cgc ggc cgc cat taa gac tta aat tag ga | deletion of *tfs4* (NotI) |
| **SR34** | Tfs4 | antisense | gat ccc gcg gat aat ctc caa att tta gag | deletion of *tfs4* (SacII) |
| **SR35** | Tfs3 | antisense | gat ccc gcg gca ctt tca tac tat att taa | deletion of *tfs3* (SacII) |
| **SR42** | *rpsL-erm* | sense | cag cat cga tat gct tta taa cta tgg att | amplification of *rpsL-erm-*cassette (ClaI) |
| **SR43** | *rpsL-erm* | antisense | gcg gta cct tac tta tta aat aat tta tag | amplification of *rpsL-erm*-cassette (KpnI) |
| **SR48** | *Tfs4* |  | gat cct cga gta ttg gtg ggc att gcc aat | amplification  *tfs4* internal |
| **SR49** | *Tfs4* |  | gat cat cga tta ttt atc cga gtg ttt atc | amplification  *tfs4* internal |
| **SR53** | *cat*_GC_ | sense | gat cgg atc ccc tac agg tct cac aca tca | cat_GC_-cassette between *orf01* and *orf11* in pHel12 (BamHI) |
| **SR54** | *cat*_GC_ | antisense | gat cgg atc ccc aaa ctt tcc cta tgg gca | cat_GC_-cassette between *orf01* und *orf11* in pHel12 (BamHI) |
| **SR59** | *aphA-3* | antisense | gat cgt cga cag cga acc att tga ggt gat ag | cloning of *aphA-3* cassette from pHel3 (SalI) |
| **SR60** | *aphA-3* | sense | gat cgg tac ccg ggg atc ttt tag aca tct | cloning of *aphA-3* cassette from pHel3 (KpnI) |
| **SR73** | *cat*_GC_ | sense | gag ctg gtg ata tgg gat ag | sequencing primer within cat_GC_-cassette |
| **SR74** | *cat*_GC_ | antisense | ctc cag agc gat gaa aac gt | sequencing primer within cat_GC_-cassette |
| **SR79** | *orf12M* | antisense | gat cgg atc ctt tgt cct agt atc taa att | deletion of *orf12M* (BamHI) |
| **SR80** | *orf12M* | sense | gat cgg atc cca gat gca tgc taa cta gac | deletion of *orf12M* (BamHI) |
| **SR99** | *orf4M* | sense | ctc gag gtg tta tct aca aaa tct ac | amplification of *orf4M* from pHel4 (XhoI) |
| **SR100** | *orf4M* | antisense | gtc gac gct gat ttt ttg aca gaa aaa t | amplification of *orf4M* from pHel4 (SalI) |
| **SR105** | *orf12M* | sense | gat cga atc cgt gat gga tta taa aga att | amplification of *orf12M* (EcoRI) |
| **SR106** | *orf12M* | antisense | gat cgt cga cgc tga ttt ttt gac aga aaa at | Amplification of *orf12M* (SalI) |
| **WS542** | *Tfs3* |  | acg cct cga gca tat gga ctt aga caa act caa ag | amplification  *tfs3* internal |
| **WS355** | *Tfs3* |  | acc ggt cga caa att gca ttt tca tgc g | amplification  *tfs3* internal |
| **WS418** |  |  | acc ggt cga cta aaa aac att tca tat ctc | amplification  *cag-PAI* internal |
| **Hp542f** |  |  | ggg gat ccg tcg acc cga tct tag ggt att aac | amplification  *cag-PAI* internal |
|  |  |  |  |  |
